# High-Throughput, Machine Learning–Based Quantification of Steatosis, Inflammation, Ballooning, and Fibrosis in Biopsies From Patients With Nonalcoholic Fatty Liver Disease

**DOI:** 10.1016/j.cgh.2019.12.025

**Published:** 2020-08

**Authors:** Roberta Forlano, Benjamin H. Mullish, Nikolaos Giannakeas, James B. Maurice, Napat Angkathunyakul, Josephine Lloyd, Alexandros T. Tzallas, Markos Tsipouras, Michael Yee, Mark R. Thursz, Robert D. Goldin, Pinelopi Manousou

**Affiliations:** ∗ Liver Unit, Division of Digestive Diseases, Department of Metabolism, Digestion and Reproduction, London, United Kingdom; § Centre for Pathology, Department of Medicine, London, United Kingdom; ¶ Department of Endocrinology, Healthcare NHS Trust and Imperial College, London, United Kingdom; ‡ Department of Informatics and Telecommunications, University of Ioannina, Greece; ‖ Department of Engineering Informatics and Telecommunications, University of Western Macedonia, Greece

**Keywords:** NASH, NASH CRN, Diagnostics, Artificial Intelligence, Ballooning%, ballooning percentage, CPA, collagen proportionate area, Fat%, fat percentage, FU, follow-up evaluation, ICC, interclass correlation coefficient, Inflammation%, inflammation percentage, IQR, interquartile range, JTT, Jonckheere–Terpstra test, NAFLD, nonalcoholic fatty liver disease, NAS, nonalcoholic fatty liver disease activity score, NASH, nonalcoholic steatohepatitis, NASH CRN, Nonalcoholic Steatohepatitis Clinical Research Network

## Abstract

**Background & Aims:**

Liver biopsy is the reference standard for staging and grading nonalcoholic fatty liver disease (NAFLD), but histologic scoring systems are semiquantitative with marked interobserver and intraobserver variation. We used machine learning to develop fully automated software for quantification of steatosis, inflammation, ballooning, and fibrosis in biopsy specimens from patients with NAFLD and validated the technology in a separate group of patients.

**Methods:**

We collected data from 246 consecutive patients with biopsy-proven NAFLD and followed up in London from January 2010 through December 2016. Biopsy specimens from the first 100 patients were used to derive the algorithm and biopsy specimens from the following 146 were used to validate it. Biopsy specimens were scored independently by pathologists using the Nonalcoholic Steatohepatitis Clinical Research Network criteria and digitalized. Areas of steatosis, inflammation, ballooning, and fibrosis were annotated on biopsy specimens by 2 hepatobiliary histopathologists to facilitate machine learning. Images of biopsies from the derivation and validation sets then were analyzed by the algorithm to compute percentages of fat, inflammation, ballooning, and fibrosis, as well as the collagen proportionate area, and compared with findings from pathologists’ manual annotations and conventional scoring systems.

**Results:**

In the derivation group, results from manual annotation and the software had an interclass correlation coefficient (ICC) of 0.97 for steatosis (95% CI, 0.95–0.99; *P* < .001); ICC of 0.96 for inflammation (95% CI, 0.9–0.98; *P* < .001); ICC of 0.94 for ballooning (95% CI, 0.87–0.98; *P* < .001); and ICC of 0.92 for fibrosis (95% CI, 0.88–0.96; *P* = .001). Percentages of fat, inflammation, ballooning, and the collagen proportionate area from the derivation group were confirmed in the validation cohort. The software identified histologic features of NAFLD with levels of interobserver and intraobserver agreement ranging from 0.95 to 0.99; this value was higher than that of semiquantitative scoring systems, which ranged from 0.58 to 0.88. In a subgroup of paired liver biopsy specimens, quantitative analysis was more sensitive in detecting differences compared with the nonalcoholic steatohepatitis Clinical Research Network scoring system.

**Conclusions:**

We used machine learning to develop software to rapidly and objectively analyze liver biopsy specimens for histologic features of NAFLD. The results from the software correlate with those from histopathologists, with high levels of interobserver and intraobserver agreement. Findings were validated in a separate group of patients. This tool might be used for objective assessment of response to therapy for NAFLD in practice and clinical trials.

**What You Need to Know**

**Background**

Histologic scoring systems are subjective and do not reproducibly identify patients with nonalcoholic fatty liver disease (NAFLD). Automated techniques for liver biopsy analysis have required expensive reagents and specialized equipment.

**Findings**

We developed and validated a user-friendly, high-throughput, automated technique for quantitation of fat, inflammation, ballooning, and collagen in liver biopsy specimens. An algorithm was devised using machine learning and developed using liver biopsy specimens from patients with NAFLD. Results correlated with those from histopathologists and there was a high level of reproducibility among users. Results also were more sensitive in detecting changes compared with traditional scores in a cohort of paired liver biopsy specimens.

**Implications for patient care**

Automated quantitation of features of liver biopsy specimens might support histopathologists and increase reproducibility in detection of histologic features of NAFLD. This tool might be developed to determine responses to therapeutic agents in practice and clinical trials.

Nonalcoholic fatty liver disease (NAFLD) is an increasing cause of chronic liver disease worldwide, with an estimated global prevalence of approximately 25%. It is associated closely with type 2 diabetes and the metabolic syndrome, with the increasing incidence of the disease closely reflecting population trends toward increasing levels of obesity,^1^ to the extent that NAFLD is now the second most common etiology of liver disease requiring liver transplantation in the United States.^2^

Liver biopsy remains the reference standard for the diagnosis and staging of NAFLD, with the Nonalcoholic Steatohepatitis Clinical Research Network (NASH CRN) Scoring System commonly used to stage disease severity.^3^ This semiquantitative system consists of a set of scores allocated by the pathologists for each of 4 key histologic features: steatosis (0–3), lobular inflammation (0–3), hepatocyte ballooning (0–2), and fibrosis (0–4). The first 3 features have their respective scores summed to generate the NAFLD Activity Score (NAS) (0–8), and the fibrosis score is allocated based on an assessment of specific architectural patterns of fibrosis.

The NASH CRN scoring system was developed by a group of 9 expert academic liver pathologists, between whom there was a high level of agreement.^4^ However, other studies have identified poor reproducibility in the assessment of key features of NASH, even among specialist pathologists,^5^ with even lower reproducibility between general pathologists.^6^ This lack of consistency and objectivity is a concern, particularly in the context of NAFLD clinical trials using histologic end points. More specifically, the resolution of NASH without worsening of fibrosis, or the improvement of fibrosis without resolution of NASH, are commonly used criteria in current NAFLD trials, and the need for rapidly assessed, objective, and reproducible end points currently is unmet.

For more than a decade, a range of morphometric techniques and computerized image analysis programs have been developed with the aim of providing more reproducible results for grading histologic features in liver disease,^7^ and principally steatosis^8^ and fibrosis.^9^^,^^10^ Such methods consistently show clear advantages related to reproducibility and objectivity over semiquantitative scoring, but none of them is presently in clinical use because most require high-resolution images and often require specialized equipment.^11^ Furthermore, to our knowledge, very few studies have attempted a quantitative assessment of ballooning and inflammation in NAFLD.^12^^,^^13^ A recent consensus document from the Case Definitions Working Group of the Liver Forum recognized the potential role of quantitation as an entry criterion to drug trials within the field.^14^

This study’s primary aim was to develop and validate a high-throughput, fully automated, machine learning–based system for the quantitation of all 4 key histologic features contributing to the NASH CRN score, using liver biopsy specimens obtained from patients with NAFLD.

## Materials and Methods

### Study Population

We retrospectively assessed all consecutive patients with biopsy-proven NAFLD followed-up at the Liver Unit of St. Mary’s Hospital (Imperial College Healthcare NHS Trust, London, United Kingdom) from January 2010 to December 2016. The study population therefore was divided into 2 subgroups: the derivation cohort (including patients who underwent liver biopsy from January 2010 to December 2012) and the validation cohort (including those who had the procedure from January 2013 to December 2016).

At the time of the liver biopsy, a full range of clinical parameters was recorded. Exclusion criteria were the use of steatogenic drugs, excess alcohol consumption (>14 units/wk), as well as comorbidities.

### Liver Histology

Liver biopsies were performed using the Menghini^15^ technique. Further details are available in the Supplementary Methods section. All 4 features were annotated manually in the images of liver biopsy specimens from the derivation group by either one or the other of the expert hepatobiliary pathologists (working independently of each other) to allow training of the machine learning algorithm used to perform the automated image analysis. Finally, the image analysis developed from the derivation group was used for the quantitation of all 4 features in images of the liver biopsy specimens from the validation cohort.

### Image Analysis for Steatosis, Hepatocyte Ballooning, and Inflammation

**Figure 1:**
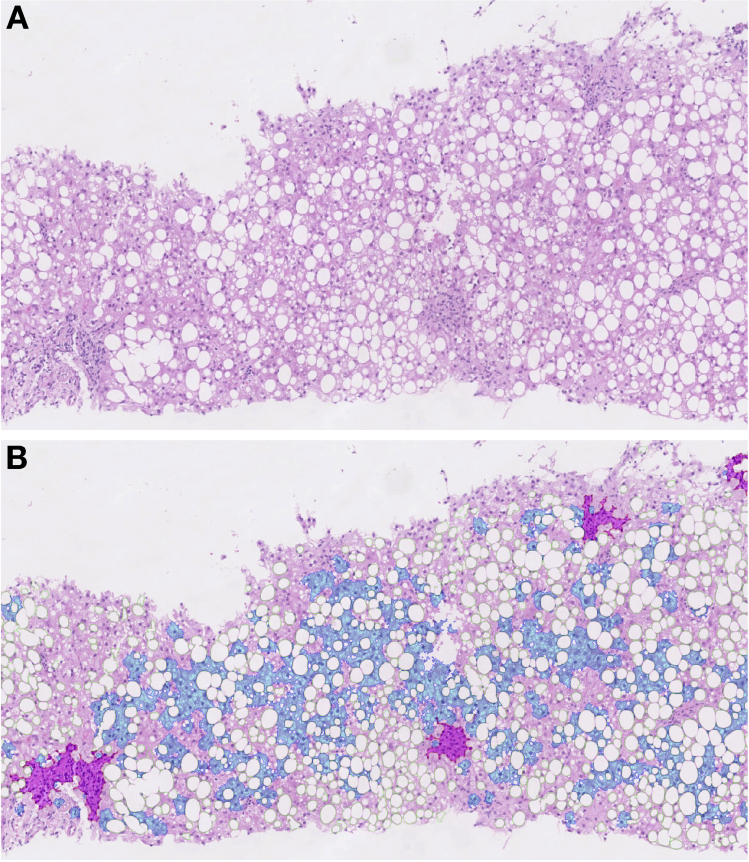
Image analysis for quantitation of steatosis, inflammation, and ballooning. (*A*) Magnified image of a liver biopsy specimen stained in H&E and scored as steatosis grade 3 (moderate, ≥66%), lobular inflammation score of 1 (≤2 foci), and ballooning score of 1 (few ballooned cells). (*B*) Results of image analysis were as follows: fat was 30.9% (in green), inflammation was 3.4% (in purple), and ballooning was 10.8% (in blue).

The proposed methodology for quantitation of these features engaged machine learning techniques with conventional image processing methods. Full details are provided in the Supplementary Methods section. The results of the quantitation are expressed as the percentage of fat (fat%), percentage of inflammation (inflammation%), and percentage of ballooning (ballooning%). An example of the output from the machine learning algorithm is shown in Figure 1.

### Image Analysis for Fibrosis

**Figure 2:**
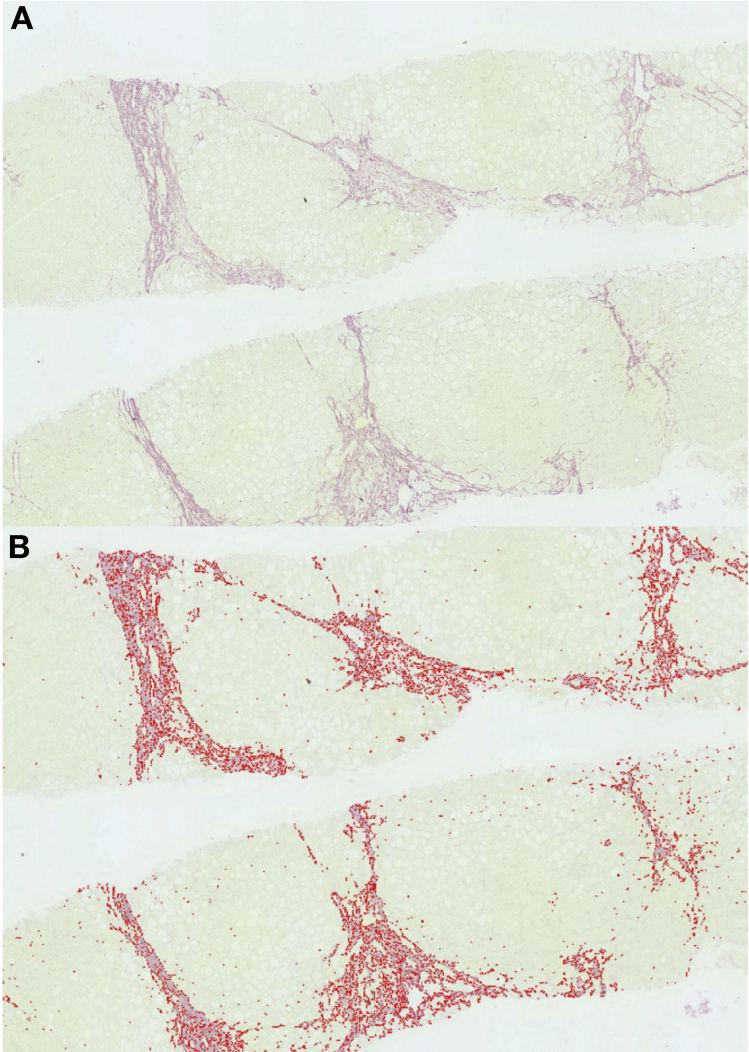
Image analysis for quantitation of fibrosis. (*A*) Image of a liver biopsy stained in Sirius red and scored as fibrosis stage 4 as per the Nonalcoholic Steatohepatitis Clinical Research Network scoring system. (*B*) Result of image analysis showing a collagen percentage area of 22.5%.

The proposed methodology to quantify fibrosis already has been validated in patients with chronic hepatitis C infection.^16^ Briefly, it provides a fully automated image analysis of liver biopsy specimens to extract the collagen proportional area (CPA) (Figure 2). This algorithm also includes a final step that allows the user to remove any structural collagen (eg, collagen from large portal tracts, blood vessel wall, and capsule) from the final quantitation of CPA, similar to the methodology used in comparable studies.^17^

### Statistical Analysis

Statistical analysis and details regarding the analysis of reproducibility are provided in the Supplementary Methods section.

## 1 Results

### Study Population

A total of 246 consecutive patients with biopsy-proven NAFLD (190 with NASH and 56 with simple steatosis) were evaluated retrospectively. The first 100 patients were included in the derivation cohort and the following 146 patients were included in the validation cohort.

Clinical characteristics of included patients are shown in Tables 1 and 2, respectively.

**Table 1:** Clinical, Demographic, and Biochemical Characteristics of the Study Population

	Study population (n = 246), N (%)	Derivation cohort (N = 100), N (%)	Validation cohort (N = 146), N (%)	*P* value^a^
Male sex	169 (69)	65 (65)	104 (71)	.23
Ethnic group				
White non-Hispanic	112 (46)	50 (50)	62 (42)	.08
White Hispanic	16 (6)	6 (6)	10 (6)	.78
Asiatic	69 (28)	24 (24)	45 (31)	.23
Black	49 (20)	19 (19)	30 (21)	.67
Type 2 DM	121 (49)	41 (41)	80 (54)	.35
Arterial hypertension	110 (44)	31 (31)	79 (54)	**.001**
Dyslipidemia	132 (53)	54 (54)	78 (53)	.12

	Median (range)	Median (range)	Median (range)	
Age, *y*	51 (19–77)	53 (21–77)	50 (19–75)	.19
BMI, *kg/m*^*2*^	29.2 (21–45)	29.3 (21–44.7)	28.9 (22–45)	.17
PLT, *10*^*9*^*/L*	219 (55–387)	214 (68–345)	225 (55–387)	.21
ALT, *IU/L*	63 (10–257)	58 (10–246)	64 (35–257)	.15
AST, *IU/L*	76 (18–367)	70 (21–367)	79 (18–312)	.08
Total cholesterol, *mmol/L*	4.5 (1–8)	4.6 (1.8–7.7)	4.2 (1–8)	.16
Triglycerides, *mmol/L*	3.8 (1.3–7.3)	3.6 (1.3–7.2)	4 (2–7.3)	.26
HDL, *mmol/L*	1.7 (0.3–3.3)	1.9 (0.5–2.9)	1.8 (0.3–3.3)	.61
LDL, *mmol/L*	3.55 (1.3–6.8)	3.1 (1.3–5.8)	3.6 (1.5–6.8)	.11
HbA1c, *mmol/L*	43 (21–113)	46 (25–85)	41 (21–113)	.98
Ferritin, *μg/L*	146 (6–912)	166 (8–912)	143 (25–844)	.12

ALT, alanine aminotransferase; AST, aspartate aminotransferase; BMI, body mass index; HbA1c, glycated hemoglobin; HDL, high-density lipoprotein; LDL, low-density lipoprotein; PLT, platelet; type 2 DM, type 2 diabetes mellitus.

a *P* value for the difference between the derivation group and the validation group.

**Table 2:** Histologic Characteristics of the Study Population and the Derivation and Validation Cohorts

	Study population (N = 246), N (%)	Derivation cohort (N = 100), N (%)	Validation cohort (N = 146), N (%)	*P* value^a^
Steatosis				.44
Grade 1	70 (28)	26 (26)	44 (30)	
Grade 2	139 (57)	58 (58)	81 (56)	
Grade 3	37 (15)	16 (16)	21 (14)	
Lobular inflammation				.18
Score 0	41 (17)	10 (10)	31 (21)	
Score 1	163 (67)	73 (73)	90 (62)	
Score 2	38 (15)	16 (16)	22 (15)	
Score 3	4 (1)	1 (1)	3 (2)	
Ballooning				.41
Score 0	56 (23)	14 (14)	42 (29)	
Score 1	116 (47)	54 (54)	62 (42)	
Score 2	74 (30)	32 (32)	42 (29)	
Fibrosis				.3
0	24 (10)	9 (9)	15 (10)	
1	67 (27)	20 (20)	47 (32)	
1a	27 (11)	10 (10)	17 (11.4)	
1b	3 (1)	2 (2)	1 (0.6)	
1c	37 (15)	8 (8)	29 (19)	
2	40 (16)	21 (21)	19 (13)	
3	82 (34)	35 (35)	47 (33)	
4	33 (13)	15 (15)	18 (12)	

a *P* value for the difference between the derivation and validation groups.

### Derivation Cohort

#### Steatosis assessment

**Figure 3:**
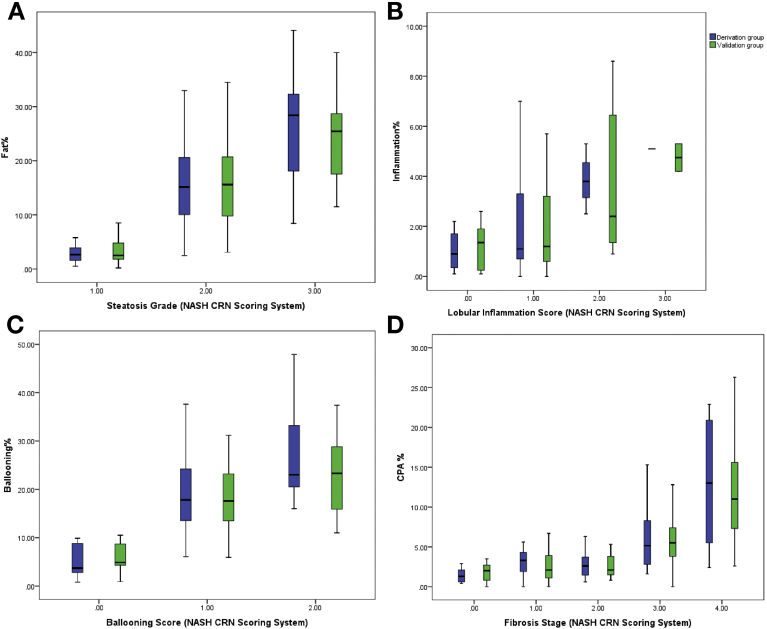
Nonalcoholic Steatohepatitis Clinical Research Network (NASH CRN) scoring system against quantitation in the derivation and validation groups. (*A*) Fat percentage (fat%) and steatosis grade. (*B*) Inflammation percentage (inflammation%) and inflammation score. (*C*) Ballooning percentage (ballooning%) and ballooning score. (*D*) Collagen percentage area (CPA%) and fibrosis stage.

In the derivation group, the median percentage of fat for each grade was as follows: 2.6% (interquartile range [IQR], 1.7%–3.8%) for grade 1 (5%–33%); 15.1% (IQR, 10.1%–20.1%) for grade 2 (34%–67%); and 28.4% (IQR, 20.2%–31.9%) for grade 3 (>67%) (Table 3). The Spearman correlation between the percentage of fat and steatosis grade was strong (Rho = 0.66; *P* < .001), but with considerable overlap between the groups (Figure 3*A* and Supplementary Table 1).

**Table 3:** Results of the Image Analysis for the Derivation and Validation Cohorts

	Derivation cohort, median (IQR)	Validation cohort, median (IQR)	*P* value
Steatosis	Fat%		
Grade 1	2.65 (1.7–3.8)	2.5 (1.8–4.8)	.18
Grade 2	15.1 (10.1–20.1)	15.6 (9.8–20.7)	.42
Grade 3	28.4 (20.2–31.9)	26.1 (22.2–30.5)	.61
Lobular inflammation	Inflammation%		
Score 0	0.9 (0.35–1.7)	1.3 (0.2–1.7)	.82
Score 1	1.1 (0.7–3.3)	1.2 (0.6–3.2)	.91
Score 2	3.8 (3.15–4.17)	2.85 (3.3–7.7)	.36
Score 3	5.1 (N/A)	4.7 (4.4–5)	.24
Ballooning	Ballooning%		
Score 0	4.9 (4.3–8.7)	6.7 (2.8–8.8)	.2
Score 1	17.8 (13.5–24)	17.6 (13.5–22.8)	.79
Score 2	23 (20.2–32.3)	23.3 (15.9–28.8)	.37
Fibrosis	CPA		
0	1.3 (0.6–2)	2 (0.9–2.6)	.1
1	2.3 (1.9–4.3)	2.1 (1.1–3.7)	.87
2	2.4 (2.6–3.6)	2.1 (1.5–3.8)	.9
3	5.1 (2.8–8.2)	5.5 (3.8–7.4)	.11
4	13 (5.5–20.9)	11.1 (7.6–16.6)	.19

ballooning%, ballooning percentage; CPA, collagen proportionate area; Fat%, fat percentage; inflammation%, inflammation percentage; IQR, interquartile range.

The fat% derived from the automated quantitation then was compared with the ratio obtained by the manual annotations of the histopathologists. There was excellent concordance between manual annotations and automatic measurements, with an interclass correlation coefficient (ICC) of 0.97 (95% CI, 0.95–0.99; *P* < .001).

#### Inflammation assessment

In the derivation group, the median percentage of inflammation for each score was as follows: 0.9% (interquartile range [IQR], 0.3%–1.7%) for a score of 0; 1.1% (IQR, 0.7%–3.3%) for a score of 1, 3.8% (IQR, 3.15%–4.17%) for a score of 2; and 5.1% for a score of 3 (Table 3). The Spearman correlation between inflammation% and inflammation score was significant (Rho = 0.36; *P* < .001) and the relation was linear (Jonckheere–Terpstra test [JTT] test z = 4.2; *P* < .001). A significant overlap was evident between the percentage of inflammation and inflammation scores (Figure 3*B* and Supplementary Table 1).

The percentage of inflammation derived from the automated quantitation then was compared with the ratio obtained by the manual annotations of the histopathologists. There was excellent concordance between manual annotations and automatic measurements, with an ICC of 0.96 (95% CI, 0.9–0.98; *P* < .001).

#### Ballooning assessment

In the derivation cohort, the median percentage of ballooning for each score was as follows: 4.9% (IQR, 4.3%–8.7%) for a score of 0, 17.8% (IQR, 13.5%–24%) for a score of 1; and 23% (IQR, 20.2%–32.3%) for a score of 2 (Table 3). The Spearman correlation between the percentage of ballooning and ballooning score was statistically significant (Rho = 0.52; *P* < .001) and the relation was linear (JTT test z = 4.4; *P* < .001). There was a significant overlap between ballooning% and ballooning scores (Figure 3*C* and Supplementary Table 1).

The percentage of ballooning derived from the automated quantitation then was compared with the ratio obtained by the manual annotations of the histopathologists. There was excellent concordance between manual annotations and automatic measurements, with an ICC of 0.94 (95% CI, 0.87–0.98; *P* < .001).

#### Fibrosis assessment

In the derivation group, the median CPA for each stage was as follows: 1.3% (IQR, 0.6%–2%) for stage 0; 2.3% (IQR, 1.9%–4.3%) for stage 1; 2.4% (IQR, 1.6%–3.6%) for stage 2; 5.1% (IQR, 2.8%-8.2%) for stage 3; and 13% (IQR, 5.5–20.9) for stage 4 (Table 3). The Spearman correlation between CPA and fibrosis stage had a Rho value of 0.57 (*P* = .01). Significant overlap was evident between early stages of fibrosis (Figure 3*D* and Supplementary Table 1).

CPA derived from the automated quantitation then was compared with the ratio obtained by the manual annotations of the histopathologists. There was excellent concordance between manual annotations and automatic measurements, with an ICC of 0.92 (95% CI, 0.88–0.96; *P* < .001).

### Validation of Image Analysis in the Validation Cohort

In the validation cohort, the median percentage of fat was 2.5% (IQR, 1.8%–4.8%) for grade 1, 15.6% (9.8%–20.7%) for grade 2, and 26.1% (IQR, 22.2%–30.5%) for grade 3. There was no difference between the derivation and validation groups in terms of the median percentage of fat (Table 3).

The median percentage of inflammation was 1.3% (IQR, 0.2%–1.7%) for a score of 0; 1.2% (IQR, 0.6%–3.2%) for a score of 1; 2.85% (IQR, 3.3%–7.7%) for a score of 2; and 4.7% for a score of 3 (IQR, 4.4%–5%). There was no difference between the derivation and validation groups in terms of median percentage of inflammation (Table 3).

The median percentage of ballooning was 6.7% (IQR, 2.8%–8.8%) for a score of 0; 17.6% (IQR, 13.5%–22.8%) for a score of 1; and 23.3% (IQR, 15.9%–28.8%) for a score of 2. There was no difference between the derivation and validation groups in terms of the median percentage of ballooning (Table 3).

The median percentage of CPA was 2% (IQR, 0.9%–2.6%) for a stage of 0; 2.1% (IQR, 1.1%–3.7%) for a stage of 1; 2.1% (IQR, 1.5%–3.8%) for a stage of 2; 5.5% (IQR, 3.8%–7.4%) for a stage of 3; and 11.1% (IQR, 7.6%–16.6%) for a stage of 4. There was no difference between the derivation and validation groups in terms of CPA (Table 3).

Binary logistic regression was used to generate a variable that combined the percentage of fat, ballooning, and inflammation for predicting the presence of NASH (NAS score, ≥5):

combined variable = 0.058 ∗ (fat%) + 0.079 ∗ (ballooning%) + 0.485 ∗ (inflammation%) – 3.882.

**Supplementary Figure 1:**
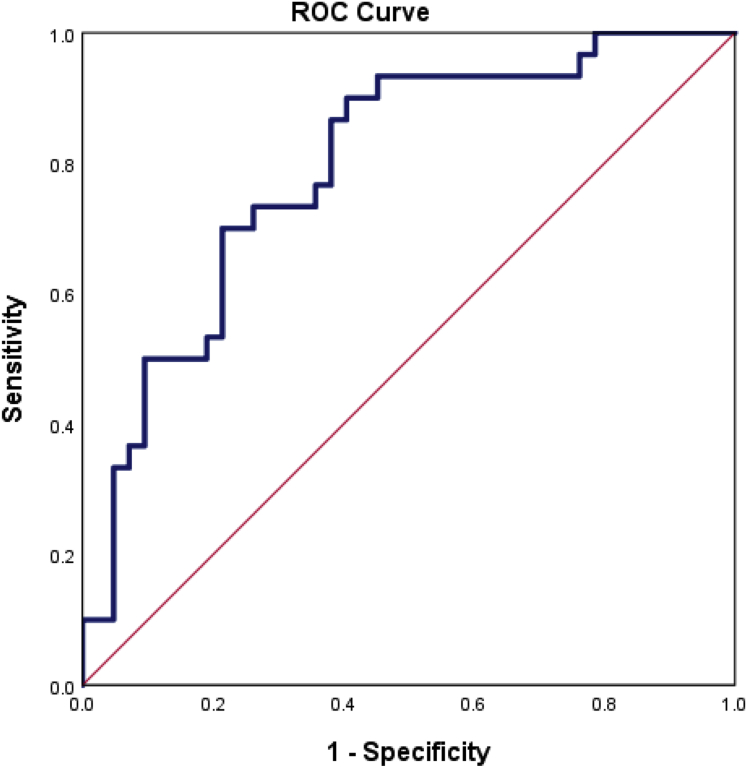
The area under the receiver operating characteristic curve (ROC) curve for the variable combining the percentage of fat, percentage of ballooning, and percentage of inflammation for diagnosing nonalcoholic steatohepatitis (nonalcoholic fatty liver disease activity score, ≥5).

The area under the receiver operating characteristic curve of such variables for diagnosing NASH (NAS score, ≥5) was 0.802 (95% CI, 0.68%–0.89%; *P* = .001) (Supplementary Figure 1). A cut-off value of 0.31 showed a sensitivity of 80%, a specificity of 62%, a positive predictive value of 60%, and a negative predictive value of 72%.

The areas under the receiver operating characteristic curves of CPA for diagnosing fibrosis F ≥ F2, F ≥ F3, and F4 were 0.72 (95% CI, 0.66–0.8; *P* < .001), 0.82 (95% CI, 0.76–0.88; *P* < .001), and 0.89 (95% CI, 0.82–0.95; *P* < .001), respectively, with the best cut-off values of 2.05%, 3.1%, and 8.1%, respectively (Supplementary Table 2).

### 1.1 Reproducibility

In the whole population, using automated quantitation, intraobserver and interobserver agreement was excellent compared with the NASH CRN scoring system. Full details are shown in Supplementary Table 3.

### Paired Biopsy Specimens

**Supplementary Figure 2:**
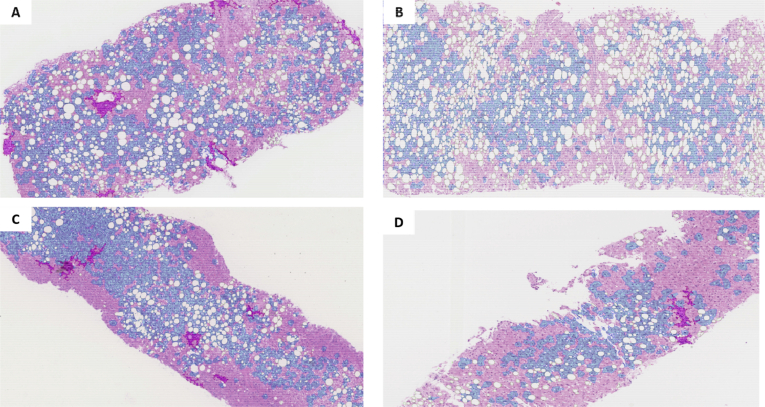
Analysis of the percentage of fat, inflammation, and ballooning in paired liver biopsy specimens. (*A* and *B*) Paired liver biopsy specimens in a patient who gained weight in a time interval of 24 months. (*A*) At the baseline liver biopsy, the Nonalcoholic Steatohepatitis Clinical Research Network (NASH CRN) scoring system was steatosis grade 2 (33%–66%), inflammation score of 1 (<2 foci), ballooning score of 2 (many ballooned cells), and the percentage of fat (fat%) was 19.8%, the percentage of inflammation (inflammation%) was 1.9%, and the percentage of ballooning (ballooning%) was 24.5%. (*B*) In the follow-up liver biopsy, the NASH CRN scoring system was steatosis grade 2 (33%–66%), inflammation score if 1 (<2 foci), ballooning score of 1 (few ballooned cells), and fat% was 30.5%, inflammation% was 0.5%, and ballooning% was 18.5%. (*C* and *D*) Paired liver biopsy specimens in a patient who lost weight during a time interval of 60 months. At the baseline liver biopsy, the NASH CRN scoring system was steatosis grade 2 (33%–66%), inflammation score of 1 (<2 foci), ballooning score of 1 (few ballooned cells), and fat% was 13.5%, inflammation% was 1.44%, and ballooning% was 32%. In the follow-up liver biopsy, the NASH CRN scoring system was steatosis grade of 1 (<33%), inflammation score of 1 (<2 foci), ballooning score of 1 (few ballooned cells), and Fat% was 6.49%, inflammation% was 1.28%, and ballooning% was 17.6%.

**Supplementary Figure 3:**
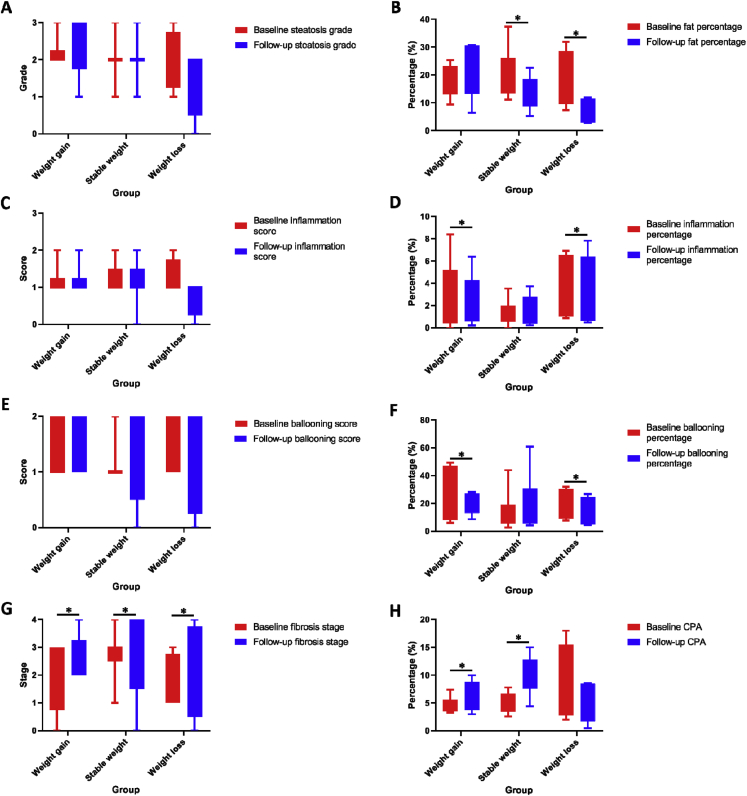
Comparison of key features from paired liver biopsy specimens, as assessed by the NASH-CRN scoring system and automated quantitation. (*A*) Steatosis grade; (*B*) fat percentage; (*C*) inflammation score; (*D*) inflammation percentage; (*E*) ballooning score; (*F*) ballooning percentage; (*G*) fibrosis stage; and (*H*) collagen proportionate area. The Mann–Whitney test was used to compare pre- and post-biopsies in all cases. ∗*P* < 05.

**Supplementary Figure 4:**
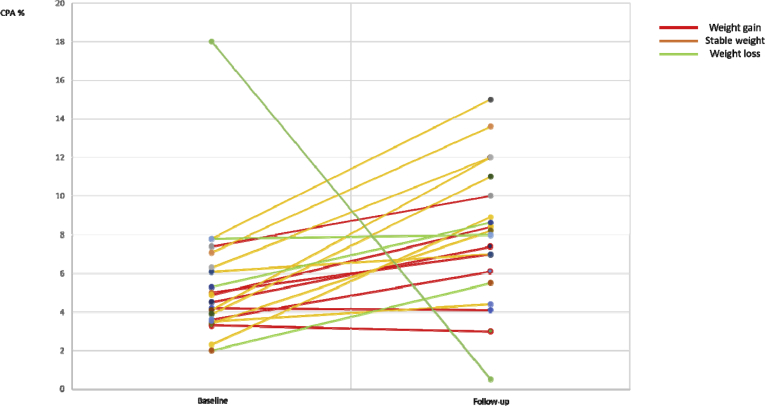
Analysis of the collagen proportionate area (CPA) in paired biopsy specimens. Differences in CPA in follow-up liver biopsy specimens compared with baseline. Patients were divided into 3 groups: those with weight gain, those with stable weight, and those with weight loss.

A subset of 20 patients underwent paired liver biopsies, with a median time interval of 45 months (range, 15–88 mo) between biopsies. The repeated liver biopsy was performed for clinical reasons (ie, to restage NAFLD). Of note, 7 patients reported significant weight gain, 9 reported stable weight, and 4 reported significant weight loss. The changes in the 4 histologic features were analyzed in each of the 3 groups (Supplementary Figure 2, Supplementary Figure 3, and 4).

#### Patients with weight gain

Overall, the median steatosis grade was 2 at baseline and 3 at follow-up evaluation (FU) (*P* = .58), with a Δsteatosis grade of +0.5. The median fat% was 19.25% at baseline and 23.43% at FU (*P* = .48), with a median Δfat% of +1.77%.

The inflammation score was 1 at baseline and 1 at FU (*P* = .9), with a Δinflammation score of 0. Inflammation% was 1.23% at baseline and 1.28% at FU (*P* = .05), with a Δinflammation% of +0.4%.

The ballooning score was 1 at baseline and 2 at FU (*P* = .57), with a Δballooning score of +0.5. Ballooning% was 15.7% at baseline and 20.3% at FU (*P* = .03), with a Δballooning% of +6.25%.

The fibrosis stage was 2 at baseline and 3 at FU (*P* = .05), with Δfibrosis stage of +1. The median CPA was 4.6% and 7.5% at FU (*P* = .028), with a ΔCPA of +2.25%.

#### Patients with stable weight

Overall, the median steatosis grade was 2 at baseline and 2 at FU (*P* = .9), with a Δsteatosis grade of 0. The median fat% was 19.5% at baseline and 13.7% at FU (*P* = .05), with a median Δfat% of -6.3%.

The inflammation score was 1 at baseline and 1 at FU (*P* = .69), with a Δinflammation score of 0. The median inflammation% was 0.87% at baseline and 1.53% at FU (*P* = .12), with a median Δinflammation% of +0.12%.

The ballooning score was 1 at baseline and 1 at FU (*P* = .63), with a Δballooning score of 0. The median ballooning% was 13.4% at baseline and 19.4% at FU (*P* = .78), with a Δballooning% of +3.76%.

The fibrosis stage was 3 at baseline and 4 at FU (*P* = .02), with a Δfibrosis stage of +1. The median CPA was 4.1% at baseline and 11.5% at FU (*P* = .001), with a ΔCPA of +6.3%.

#### Patients with weight loss

Overall, the steatosis grade was 2 at baseline and 1 at FU (*P* = .12), with a Δsteatosis grade of -0.5. The median fat% was 16.5% at baseline and 10.5% at FU (*P* = .08), with a median Δfat% of -9.95%.

The inflammation score was 1 at baseline and 1 at FU (*P* = .18), with a Δinflammation score of +0.5. The median inflammation% was 3.4% at baseline and 1.6% at FU (*P* = .04), with a Δinflammation% of -0.44%.

The ballooning score was 1.5 at baseline and 1 at FU (*P* = .17), with a Δballooning score of -0.5. The median ballooning% was 19.3% at baseline and 12.35% at FU (*P* = .04), with a Δballooning% of -5.47%.

Fibrosis stage was 1.5 at baseline and 2.5 at FU (*P* = .05), with a Δfibrosis stage of +1. The median CPA was 6.55% at baseline and 6.75% at FU (*P* = .12), with ΔCPA of 1.75%.

## Discussion

Histology remains the reference standard to diagnose and stage NAFLD. In the absence of validated noninvasive markers, liver biopsy remains the only modality through which the presence of NASH may be assessed.^4^ The NASH CRN score, the widely validated histologic system for grading NASH, was not designed to replace the histopathologist’s overall assessment of disease category (eg, NASH/borderline NASH/not NASH), but rather to provide a measurable scale for use in trial end points. However, significant concerns exist regarding the reproducibility of the assessment of these histologic features between different pathologists by conventional scores.^5^^,^^6^ There also are questions about the objectivity of these techniques, as shown by the apparent significant disparities between the quantitation of fat on liver biopsy specimens made by pathologists when compared with using more objective assessment methods.^18^ In this study, we propose a technique based on image analysis and machine learning for the quantitation of all 4 key histologic features included within the NASH CRN scoring system.

The study involved 2 hepatobiliary pathologists examining biopsy specimens from a large cohort of patients with NAFLD. The cohort included patients with the full spectrum of the condition, with typical comorbidities seen in Western practice, and across a range of ethnicities.

The techniques described here require only modest computational effort, thus consuming very little time and avoiding the need to purchase specialist equipment. The machine learning software is straightforward to install on any device, and quantitation is performed usually within 2 minutes. Therefore, this technology could be applied broadly, even in nonspecialist centers. Moreover, these image analyses, through machine learning techniques, are fully automated and do not require any manual intervention in any step. This is a major advantage compared with other approaches presented in the literature requiring manual input,^7^^,^^8^^,^^19^ which have an inherent risk of introducing bias. However, it also should be appreciated that a liver biopsy in a patient with NAFLD may provide other valuable histologic information, including assessment of other potential diagnoses or features, such as iron overload.

Our study raises some important issues with the traditional reporting systems, showing a significant overlap as well as only a moderate correlation (Rho, ∼0.5) between semiquantitative scores and quantitative results. First, in the sole category in which a direct comparison of quantitation can be made (steatosis), the pathologists consistently overestimated the fat content (median values for NASH CRN stages 1–3 by quantitation were 2.5% vs 15.6% vs 26.1%, respectively), highlighting the limitation of making a quantitative assessment by visual inspection alone. Second, the inflammation score and inflammation quantitation overlapped significantly, although showing a linear relation. This may be because the inflammation score assesses the number of foci of inflammation, whereas the image analysis provides the proportional area of inflammation. Of note, our image analysis includes both lobular and portal inflammation compared with the score that provides lobular inflammation only. Further discussion about steatosis, inflammation, and ballooning% is provided in the Supplementary Discussion section.

In terms of fibrosis evaluation, the CPA increased with each fibrosis stage in an exponential rather than linear fashion, in keeping with previous reports.^17^ The Brunt et al^20^ system for reporting fibrosis, used alongside the NASH CRN score, describes architectural features rather than the quantity of collagen, and the prognostic significance has been well validated by large cohorts with long-term follow-up data.^21^, ^22^, ^23^ Interestingly, CPA also has been associated independently with clinical outcomes in NAFLD, in addition to fibrosis stage.^24^

In addition, taken together, our results raise important questions on how to use liver histology to inform end points of clinical trials. Analyzing a subgroup of paired liver biopsy specimens, we have shown that the CRN scoring system is not as sensitive in showing changes compared with quantitation of histologic features. This finding has been particularly striking in the assessment of inflammation and ballooning. Moreover, by combining the percentage of fat, ballooning, and inflammation, it was possible to diagnose NASH accurately using our algorithm; however, the gold standard for the diagnosis of NASH is based on variable combinations of semiquantitative scores in the NAS system, which still remains primarily academic rather than embedded in clinical practice. Furthermore, our quantitation software was not designed primarily to diagnose NASH, but to stage the disease more accurately. By introducing a more sensitive and reliable system, automated quantitation may provide different results in clinical trials and new insights into the pathophysiology of the disease. Moreover, we have shown that CPA increases exponentially with fibrosis stage, challenging the dogma of 1 or more stage reduction or no worsening of fibrosis as outcomes. Given the pattern we have shown, a reduction from stage 4 to stage 3 would reflect a markedly higher antifibrotic effect than from stage 2 to stage 1. Moreover, it may be that a reduction in CPA within stage 4 still may have important clinical benefits, such as risk of decompensation. This needs to be shown in more studies, but we agree with recent calls to include CPA within trial end points.^25^

Our present study shows an important limitation, which is the absence of an external validation cohort. However, we conducted an internal validation across a large cohort of patients who collectively represent the full spectrum of NAFLD severity.

In conclusion, we have developed a fast-operating and accurate automated image analysis method to quantitate steatosis, ballooning, inflammation, and fibrosis in routine histologic images of patients with NAFLD. These methodologies do not require sophisticated equipment and have shown reliable and reproducible results. Given the key role for the assessment of these features in NASH clinical trials, there is a compelling argument that these techniques should be considered for use as clinical trial end points. There is now a pressing need for related outcome data to assess their role in everyday practice.

## Figures and Tables

**Supplementary Table 1:** Semiquantitative Scores Vs Increasing Values of Quantitation

		Cases, N
Fat%	Steatosis grade, NASH CRN scoring system	
<10	Mild	61
	Moderate	36
	Severe	3
10.1–20	Mild	7
	Moderate	69
	Severe	7
20.1–30	Mild	2
	Moderate	25
	Severe	13
>30	Mild	0
	Moderate	9
	Severe	14
Inflammation%	Inflammation score, NASH CRN scoring system	
<1	Absent	19
	<2 foci	65
	2–4 foci	3
	>4 foci	0
1.1–5	Absent	22
	<2 foci	73
	2–4 foci	19
	>4 foci	1
>5	Absent	0
	<2 foci	25
	2–4 foci	14
	>4 foci	3
Ballooning%	Ballooning score, NASH CRN scoring system	
<10	Absent	37
	Few ballooned cells	19
	Many ballooned cells	7
10.1–20	Absent	18
	Few ballooned cells	44
	ballooned cells	21
20.1–30	Absent	1
	Few ballooned cells	34
	Many ballooned cells	23
>30.1	Absent	0
	Few ballooned cells	19
	Many ballooned cells	23
CPA%	Fibrosis stage, NASH CRN scoring system	
<2	Stage 0	12
	Stage 1	28
	Stage 2	20
	Stage 3	10
	Stage 4	0
2.1–5	Stage 0	10
	Stage 1	34
	Stage 2	17
	Stage 3	34
	Stage 4	0
5.1–10	Stage 0	2
	Stage 1	5
	Stage 2	3
	Stage 3	28
	Stage 4	11
>10.1	Stage 0	0
	Stage 1	0
	Stage 2	0
	Stage 3	11
	Stage 4	22

Ballooning%, percentage of ballooning; CPA%, percentage of collagen proportionate area; Fat%, percentage of fat; Inflammation%, percentage of inflammation; NASH CRN, Nonalcoholic Steatohepatitis Clinical Research Network.

**Supplementary Table 2:** AUROCs of Fat%, Inflammation%, and Ballooning% for Diagnosing NASH (NAS Score, ≥5) and AUROCs of CPA for Diagnosing F ≥ 2, F ≥ 3, and F4

Diagnosis of F ≥ 2						
CPA	2.05	0.72 (0.66–0.8)	80	46	59	70
Diagnosis of F ≥ 3						
CPA	3.1	0.82 (0.76–0.88)	72	69	64	73
Diagnosis of F4						
CPA	8.1	0.89 (0.82–0.95)	81	78	66	95

AUROC, area under the receiver operating characteristic curve; CPA, collagen proportionate area; NAS, Nonalcoholic Fatty Liver Disease Activity Score; NASH, nonalcoholic steatohepatitis.

**Supplementary Table 3:** Interobserver and Intraobserver Agreement κ Coefficients Between Two Pathologists for the NASH CRN Scoring System and Image Analysis for the Whole Population

Histologic features	NASH CRN Scoring System		Image analysis	
	Interobserver agreement weighted κ (95% CI)^a^	Intraobserver agreement weighted κ (95% CI)^a^	Interobserver agreement ICC (95% CI)^a^	Intraobserver agreement ICC (95% CI)^a^
Steatosis	0.73 (0.52–0.94)	0.88 (0.73–0.99)	0.98 (0.95–0.99)	0.96 (0.92–0.98)
Lobular inflammation	0.68 (0.43–0.94)	0.6 (0.29–0.9)	0.99 (0.97–0.99)	0.99 (0.98–0.99)
Ballooning	0.6 (0.3–0.89)	0.58 (0.3–0.87)	0. 96 (0.92–0.98)	0.95 (0.788–0.98)
Fibrosis	0.69 (0.48–0.89)	0.61 (0.37–0.85)	0.98 (0.96–0.99)	0.97 (0.95–0.99)

ICC, intraclass correlation coefficient; NASH CRN, Nonalcoholic Steatohepatitis Clinical Research Network.

a *P* < .001.

## Supplementary Methods

### Liver Histology

Only cores greater than 30 mm in length and with more than 7 fixed complete portal tracts were included.^1^ Specimens were formalin-fixed and paraffin-embedded, and stained with H&E and Sirius red. Images were captured on a Hammamatsu whole slide scanner (Shizuoka, Japan). All biopsy specimens were scored independently by 1 of 2 hepatobiliary pathologists referring to the NASH CRN scoring system. Both pathologists were experts in liver histology, each with more than 20 years of experience in reporting on liver biopsy specimens. If either pathologist had uncertainty about scoring a particular histologic feature, they reviewed the specimen together with each other and collectively agreed on a final score by consensus. Images were captured on a Hammamatsu whole slide scanner using a 20× objective lens in Nanozoomer digital pathology image format. Images of liver biopsy specimens stained in H&E then were exported into JPEG format after a 20× magnification, using NDP.view Nanozoomer (Hamamatsu City, Japan) viewer software. Similarly, images of liver biopsy specimens stained in Sirius Red were exported into a JPEG format after a 2× magnification.

### Methodology for Image Quantitation

The tool for image analysis and quantitation is performed in 4 stages, collectively providing the steatosis, inflammation, and ballooning ratio compared with the core of the biopsy specimen. Therefore, 4 different areas are calculated (ie, tissue area, accumulated area of fat droplets, inflammation, and ballooned cells) as part of the corresponding algorithm stages. Machine learning–based techniques have been used in 2 steps during the methodology. Depending on the features of each region of the image, a clustering algorithm is applied to detect the tissue (stage 1) and to differentiate normal hepatocytes vs ballooned cells (stage 4).

#### Stage 1

H&E biopsy images have a high-intensity background, whereas liver tissue has a deep red color. Images were colored in red-green-blue color space, so that 3 channels (red, green, and blue) could be used for visualization. To identify tissue regions in the image, the method separates the pixels of tissue from background pixels using clustering techniques. In this way, all the pixels of the image are grouped into 2 separate clusters; namely, a cluster for tissue and a cluster for background. Specifically, the first stage uses the K-means algorithm, taking into account the color (ie, 3 intensity values ranging from 0 to 255) of each pixel for grouping. For both clusters, the method initially defines a color centroid (a center point of intensity values), to compare it with the color of each pixel. During K-means execution, an iterative procedure assigns each pixel of the image either to the tissue cluster or to the background cluster, based on the minimum color distance with the centroids. In each iteration of the algorithm, the centroids are reconsidered according to the color of the members (pixels) of the cluster. The iteration stops when the color centroids are stabilized for 2 consecutive iterations. At the end of the algorithm execution, tissue pixels have been identified, and the tissue area is calculated.

#### Stage 2

Once the tissue region has been identified, we attempt to detect all white regions in the core. Image processing techniques, focusing on the detection of circular white regions within tissue, are used. Initially, a thresholding method converts the image into binary (0 or 1 pixel values). Next, morphologic operations use a mask, with a specific shape and size to operate on that image. In our case, a circular mask was selected to recognize lipid droplets, eliminating all other structures. However, because of size variations between lipid droplets, an iterative procedure was used; in each iteration, the size of the circle into the mask was increased to match all droplet sizes. The result of this procedure was the generation of a binary image, in which pixels with a value of 1 belong to white regions in the core, and pixels with a value of 0 belong to normal/other tissue). The whole area of steatosis, divided by the whole tissue area, is computed as the fat% in the core (Figure 1).

#### Stage 3

The detection of all cell nuclei was the key focus for the rest of the analysis. After identification of the nucleus, both inflamed regions and ballooned cells could be detected. Nuclei were the darkest findings in the core, so a simple thresholding technique could separate them from the rest of the tissue. Furthermore, their location distribution in normal tissue was homogeneous, presenting similar distances, one with another. In contrast, inflammation areas presented a high density of nuclei, whereas in fatty regions and regions where there was a strong presence of ballooned cells, the density of nuclei was very low. In regions of inflammation, the nuclei were close enough, and therefore in most of the cases were joined to 1 dark object. Alternatively, they could be joined using morphologic closing with small structures. In this way, all the dark areas larger than the 1% of the whole tissue were characterized as inflammation. The calculated area of inflammation (including both portal and lobular inflammation) divided by the area of the whole tissue, is reported as the inflammation%.

#### Stage 4

Once the nuclei have been located in the image, the area around it belongs to the corresponding cell. The algorithm attempts cell isolation using only spatial information, assuming that a pixel of the image belongs to the cell of the nearest nucleus. From an algorithmic point of view, this assumption is equal to the development of a Voronoi diagram, using the centers of the nuclei as vertices. Clustering techniques then are used to separate isolated cells, which present features similar to ballooned cells or normal cells. The set of features is based on mean intensity and texture of the cell region. Specifically, a supervised clustering-based method was used to deploy knowledge about the ballooned cells from a set of different images. Two centroids (ballooned or nonballooned cells) were extracted using a set of 15 images (45,803 cells in total), so that the cells of a new testing image are assigned to the cluster with the minimum Euclidean distance. The members of the cluster, which present the highest intensity and rough texture around the nuclei, are characterized as ballooned cells. The area of that regions are accumulated to calculate the ratio for ballooning, and this is reported as the ballooning%.

### Statistical Analysis and Interobserver and Intraobserver Agreement

Numeric variables were summarized as medians, ranges, and IQRs. Specifically, ranges were used to describe biochemical variables, and IQRs were used to describe fat%, inflammation%, ballooning%, and CPA. Ordinal variables were expressed as relative frequencies. Frequencies were compared using the chi-squared test; continuous variables were compared with the Mann–Whitney *U* test. The relationship between automated percentage quantitation and semiquantitative scores were explored using the Pearson correlation coefficient (accepting methodologic limitations owing to the categoric nature of fibrosis scores and the continuous measurement of CPA scores, as previously noted^2^). Fibrosis stages 1a, 1b, and 1c were considered in 1 group (stage 1). For quantitative variables (manual annotations and image analysis results), concordance was measured using the ICC. Spearman correlation and the JTT for independent samples were used to assess the linear relationship between variables.

Binary logistic regression was used to generate a variable that combined fat%, ballooning%, and inflammation%. The area under the receiver operating characteristic curves then was used to assess the diagnostic performance of the results of quantitation. Optimal cut-off values were calculated to maximize sensitivity and specificity; for each cut-off value the positive predictive value and the negative predictive value were reported. All tests were 2-sided and a *P* value of .05 was considered significant. All statistical analysis was performed using SPSS (version 24.0; SPSS, Inc, Chicago, IL).

Regarding NASH CRN scoring, interobserver agreement was defined by 2 specialized hepatobiliary pathologists independently reviewing the same histologic images. Intraobserver agreement was assessed using 20 liver biopsy specimens randomly reassigned to 1 of the hepatobiliary pathologists (R.D.G.) for a second review. In the automated quantitation, intraobserver agreement was assessed by the same pathologist who had analyzed a particular sample for automated analysis on the initial run, running 20 randomly selected liver biopsy specimens through the algorithm for a second time. Interobserver agreement was assessed by a different pathologist from the one who had analyzed the original sample running 20 randomly selected liver biopsy samples through the algorithm for a second time.

Weighted κ were calculated to explore the agreement using the NASH CRN scoring system, whereas the ICC was calculated to explore the agreement when image analysis was used. Weighted κ and ICC can be considered equivalent measurements of agreement.^3^ A κ value or an ICC value of 0.2 to 0.39 was considered fair, 0.4 to 0.59 was considered moderate, 0.6 to 0.79 was considered substantial, and 0.8 or higher was considered perfect agreement.

## Supplementary Discussion

### Steatosis

It is of note that our algorithm defines fat percentage as a proportion of steatosis in the whole tissue area, rather than purely within hepatic parenchyma. It also should be noted that although the NAS score refers to the percentage of hepatocytes containing fat, all imaging analysis techniques, and practicing histopathologists, typically assess the actual percentage of parenchyma containing fat. However, we wished to minimize the need for manual input for our algorithm; it also is noteworthy that in other comparable studies in which nonparenchymal structures (including portal tracts) were excluded manually, pathologists still overestimated fat content.^4^

### Inflammation

Far from being a rare finding, portal inflammation has been described in up to 60% to 76% of NAFLD liver biopsy specimens with different disease stages and clinical features, with investigators arguing that it should be included in the NASH CRN scoring system. In particular, as previously noted by Brunt et al,^5^ the diagnosis of definite steatohepatitis or the absence of steatohepatitis based on the evaluation of patterns as well as individual lesions on liver biopsy specimens does not always correlate with threshold values of the semiquantitative NAS. In accordance with this, this article showed that there is a very strong correlation between the presence of portal inflammation and the diagnosis of steatohepatitis. Moreover, previous studies have shown that portal inflammation correlates with clinical features and is associated with an increased risk of progressive disease in both adult and pediatric biopsy specimens.^6^^,^^7^ As such, we believe that the quantitation of both lobular and portal inflammation may provide a more comprehensive approach.

### Ballooning

Of note, there is still no accepted gold standard for the assessment of ballooning. In this study, there was an overlap between the NASH CRN ballooning score and ballooning quantitation. This may be because pathologists rely more heavily on the qualitative features of ballooning in their assessment rather than quantity, something recognized in the more recently developed steatosis, activity, and fibrosis score but not in the NASH CRN.^8^ Whether quality (eg, size and shape of ballooned cells) or quantity has more prognostic significance has not been evaluated, and our data show that this is a vital area for further research to inform more robust and consistent scoring and risk stratification. With this methodology, we propose a reproducible assessment of ballooning based on simple criteria (analysis of the texture and of the intensity of the perinuclear cytoplasm as well as the analysis of the shape of cells) derived from expert histopathologist manual annotations and improved through machine learning.
